# Phytochemical screening and evaluation of cardioprotective activity of ethanolic extract of *Ocimum basilicum L.* (basil) against isoproterenol induced myocardial infarction in rats

**DOI:** 10.1186/2008-2231-20-87

**Published:** 2012-12-05

**Authors:** Fatemeh Fathiazad, Amin Matlobi, Arash Khorrami, Sanaz Hamedeyazdan, Hamid Soraya, Mojtaba Hammami, Nasrin Maleki-Dizaji, Alireza Garjani

**Affiliations:** 1 Department of Pharmacognosy, Faculty of Pharmacy, Tabriz University of Medical Sciences, Tabriz, Iran; 2 Student Research Committee, Tabriz University of Medical Sciences, Tabriz, Iran; 3 Department of Pharmacology and Toxicology, Faculty of Pharmacy, Tabriz University of Medical Sciences, Tabriz, Iran

**Keywords:** Myocardial infarction, *O.* basilicum, Basil, Antioxidant, Isoproterenol

## Abstract

**Background and the purpose of the study:**

The objectives of the present study were phytochemical screening and study of the effects of ethanolic extract of aerial parts of *Ocimum basilicum* (basil) on cardiac functions and histopathological changes in isoproterenol-induced myocardial infarction (MI).

**Methods:**

The leaves of the plant were extracted with ethanol by maceration and subjected to colorimetry to determine flavonoids and phenolic compounds. High-performance TLC analysis and subsequent CAMAG's TLC scanning were performed to quantify rosmarinic acid content. Wistar rats were assigned to 6 groups of normal control, sham, isoproterenol, and treatment with 10, 20, and 40 mg/kg of the extract two times per day concurrent with MI induction. A subcutaneous injection of isoproterenol (100 mg/kg/day) for 2 consecutive days was used to induce MI.

**Results:**

Phytochemical screening indicated the presence of phenolic compounds (5.36%) and flavonoids (1.86%). Rosmarinic acid was the principal phenolic compound with a 15.74% existence. The ST-segment elevation induced by isoproterenol was significantly suppressed by all doses of the extract. A severe myocardial necrosis and fibrosis with a sharp reduction in left ventricular contractility and a marked increase in left ventricular end-diastolic pressure were seen in the isoproterenol group, all of which were significantly improved by the extract treatment. In addition to *in-vitro* antioxidant activity, the extract significantly suppressed the elevation of malondialdehyde levels both in the serum and the myocardium.

**Conclusion:**

The results of the study demonstrate that *Ocimum basilicum* strongly protected the myocardium against isoproterenol-induced infarction and suggest that the cardioprotective effects could be related to antioxidative activities.

## Background

*Ocimum basilicum* (basil) is a plant from genus *Ocimum* belonged to family Lamiaceae. Essential oil of the plant is composed of interesting terpenoids and phenylpropanoids such as eugenol, methyl eugenol, citral, and methyl chavicol [1]. In Iran the plant is called "rayhan" and is being broadly used as vegetable and culinary herb and also used in folk medicine to treat various diseases including cardiovascular disorders. The leaves of *O. basilicum* are traditionally used as antispasmodic, carminative, digestive, stomachic, and tonic [2,3]. The merit of the traditional use of *O. basilicum* has been supported by some former studies from the genus *Ocimum*, providing several biologically active constituents present in the extracts and essential oils of the plants. Since, the complexity and chemical diversity of the compounds present in medicinal herbs play an important role in the discovery and development of new compounds, detecting the naturally occurring constituents in plants seems to be of value to achieve a better understanding of the relation between chemical constituents and biological properties of a medicinal plant. Concerning the prior phytochemical reports from *O. basilicum*, it mainly produces triterpenoids, polyphenols, steroids, and phenylpropanoids some of which, such as basilol, ocimol, basilimoside, rosmarinic acid, hydroxycinnamic acids, oleanolic acid, and betulinic acid, have been proved for having prominent biological properties [4,5]. Moreover, the essential oil content may vary considerably among the species of *Ocimum* according to the plants growth stage, extraction method, and climatic parameters as well as agrotechnical factors. In the same way as Vani et al. had confirmed significant differences in the proportions of volatile compounds from *O. basilicum* and *O. sanctum* in relation to the many factors that contribute to the fragrances content representing *O. basilicum* essential oil rich in methyl chavicol and the essential oil from *O. sanctum* rich in methyl eugenol [6].

Recently a considerable antihypertensive effect of *O. basilicum* extract in renovascular hypertensive rats has been reported [7]. The authors claimed that the effects of *O. basilicum* extract on blood pressure, cardiac hypertrophy, and endothelin level are consistent with an effect on endothelin-converting enzyme, and warrant further exploration. A finding has also indicated that aqueous and organic extracts of *O. basilicum* lower plasma lipid levels and thereby the extracts might be beneficial in the treatment of hyperlipidemia and atherosclerosis [8]. In addition, a study by Sharma et al. (2001) demonstrated that pre- and co-treatment with *Ocimum sanctum*, another species from the genus of *Ocimum*, exhibited significant protection against isoproterenol induced histopathological and biochemical changes [9]. The cadioprotective mechanism(s) appeared to be through modulation of various antioxidant parameters thereby improving the overall antioxidant defense of the myocardial tissue. In fact, the water and ethanol extracts of *O. basilicum* have been shown to have strong antioxidant activities [10].

Acute myocardial infarction is an important ischemic heart disease and a leading cause of morbidity and mortality worldwide. Isoproterenol is a synthetic β-adernoceptor agonist that its subcutaneous injection induces myocardial infarction in rats [11] and results in irreversible cellular damage and ultimately infarct-like necrosis [12,13]. The acute phase of myocardial necrosis and dysfunction induced by isoproterenol mimics changes in blood pressure, heart rate, electrocardiogram (ECG), and left ventricular dysfunction similar to that occurs in patients with myocardial infarction. The rat model of isoproterenol-induced myocardial infarction offers a reliable non-invasive technique for studying the effects of various potential cardioprotective agents [14]. Amongst various mechanisms proposed to explain isoproterenol induced cardiac damage, generation of highly cytotoxic free radicals through autooxidation of catecholamines has been implicated as one of the important causative factor [15]. Although *O. basilicum* is an important medicinal herb having antioxidant properties, its cardioprotective activity against isoproterenol induced myocardial infarction has not been studied. The aim of the present study was to investigate the effect of oral administration of hydroalcoholic extract of leaves of *O. basilicum* on isoproterenol induced myocardial injury. Myocardial dysfunction and histopathological changes induced by isoproterenol have been monitored and their modulation with different doses of *O. basilicum* was evaluated.

## Materials and methods

### Plant material

The aerial parts of *O. basilicum* were purchased before flowering in June from local market. The botanical identification was made by Dr. F. Fathiazad (Department of Pharmacognosy). A voucher specimen was deposited at the herbarium of the Faculty of Pharmacy, Tabriz University of Medical Sciences, Tabriz, Iran.

### Extract preparation

The fresh leaves (500 g) were chopped up and extracted with ethanol (96%; 2 L × 4) by maceration at room temperature and the solvent was removed at 40°C using a rotary evaporator. A greenish residue weighing 9.2 g was obtained and kept in air tight bottle in a refrigerator until use.

### Determination of total phenolic compound

The total content of phenolic compounds was determined with the Folin-Ciocalteau reagent [16]. The extract samples (0.5 ml of different dilutions) were mixed with Folin-Ciocalteu reagent (5 ml, 1:10 diluted with distilled water) for 5 min and 4 ml aqueous Na_2_CO_3_ (1 M) were then added. The mixture was allowed to stand for 15 min and the phenols were determined by colorimetry at 765 nm. The standard curve was prepared by 0, 50, 100, 150, 200, and 250 mg/ml solutions of gallic acid in methanol:water (50:50, v/v). Total phenolic contents were calculated as gallic acid equivalent per gram of the extract from a calibration curve preaperd with 0, 50, 100, 150, 200, and 250 μg/ml solutions of gallic acid in methanol.

### Determination of total flavonoid content

The content of flavonoids was determined using colorimetric aluminum chloride method [16]. Briefly, 0.5 ml solution of the extract were mixed with 1.5 ml of methanol, 0.1 ml of 10% aluminum chloride, 0.1 ml of 1 M potassium acetate and 2.8 ml of distilled water, and were left at room temperature for 30 min. The absorbance of the reaction mixture was measured spectrophotometrically at 415 nm. Total flavonoids contents were calculated as quercetin from a calibration curve.

### Quantitative analysis

A Preliminary HPTLC (high-performance TLC) analysis of the extract was performed on silica gel 60 F_254_ HPTLC plate (Merck) with ethylacetate, methanol, and water in the ratio of 100:13.5:10 as mobile phase and the spots were detected under UV [17]. Subsequently, quantitative analysis of rosmarinic acid as the major compound in the extract was performed using a fully automated CAMAG’s TLC scanner 3 coupled with an automatic TLC sampler 4, an automatic developing chamber 2, and a TLC Visualizer (CAMAG; Switzerland). The winCATS (Planar chromatography manager) software was used for analyzing the results of the scan and image of the plate.

### Determination of *in vitro* total antioxidant activity

1,1-diphenyl-2-picryl-hydrazil (DPPH; Sigma-Aldrich Quı^′^mica S.A., Madrid, Spain) was used to determine the free radical-scavenging potential of the ethanolic extract. IC_50_ (50% inhibitory concentrations) of the extract was calculated versus MeOH as a negative control and rutin was used as a positive control [18]. Briefly, a stock solution of the extract was prepared in MeOH to achieve the concentration of 1 mg/ml. Dilutions were made to obtain concentrations of 5 × 10^-2^, 5 × 10^-3^, 5 × 10^-4^, 5 × 10^-5^, 5 × 10^-6^ and 5 × 10^-7^ mg/ml. Two ml of each solution was added to 2 ml of DPPH solution (in MeOH; 80 g/ml). The absorbance was measured at 517 nm after 30 min of reaction at 25°C. The experiments were performed in duplicate and the average absorption was noted for each concentration.

### Animals

Male albino wistar rats (260-280 g) were used in this study. Rats were housed at constant temperature (20 ± 1.8°C) and relative humidity (50 ± 10%) in standard polypropylene cages, six per cage, under a 12-h light/dark cycle, and were allowed food and water freely. This study was performed in accordance with the Guide for the Care and Use of Laboratory Animals of Tabriz University of Medical Sciences, Tabriz-Iran (National Institutes of Health publication No 85–23, revised 1985).

### Induction of acute myocardial infarction

Isoproterenol (Sigma Co; USA) was dissolved in normal saline and injected subcutaneously to rats (100 mg/kg) for two consecutive days at an interval of 24 h to induce acute myocardial infarction. Animals were sacrificed 48 h after the first dose of isoproterenol.

### Experimental protocol

The animals were randomized into 6 groups consisting of 6 rats each. Rats in group 1 (normal control) received a subcutaneous injection of normal saline (0.5 ml) and were left untreated for the whole period of the experiment. In group 2 (sham) rats received a subcutaneous injection of normal saline and *O. basilicum* extract was given orally (40 mg/kg) two times per day for two days. Rats in group 3 (isoproterenol group) received a subcutaneous injection of isoproterenol (100 mg/kg) for two consecutive days at an interval of 24 h and normal saline as vehicle (0.5 ml) was given orally concurrent with and 8 h after each isoproterenol injection. Rats in groups 4 to 6 received a subcutaneous injection of isoproterenol (100 mg/kg) for two consecutive days at an interval of 24 h, the extract was suspended in saline and given orally (10, 20 and 40 mg/kg) concurrent with and 8 h after each isoproterenol injection.

### Hemodynamic measurements

Animals were anaesthetized by intraperitoneal injection of ketamin, xylazin, and acepromazin mixture 48 h after the first dose of isoproterenol. When the rats no longer responded to external stimuli, the systemic arterial blood pressure was recorded from a catheter inserted into the left carotid artery. A standard limb lead II ECG was monitored continuously throughout the experimental period. The mean arterial blood pressure was calculated from the systolic and diastolic blood pressure traces. The heart rate was calculated from the ECG. To evaluate the cardiac left ventricular function, a Mikro Tip catheter transducer (Millar Instruments, INC) was advanced into the lumen of the left ventricle. This helped to measure the left ventricular systolic pressure (LVSP), left ventricular end-diastolic pressure (LVEDP), maximum and minimum rates of developed left ventricular pressure (LVdP/dtmax and LVdP/dtmin) and the rate of pressure change at a fixed right ventricular pressure (LVdP/dt/P) [19]. All the parameters were continuously recorded using a Powerlab system (AD Instruments, Australia).

### Tissue weights

After the hemodynamic measurements, the animals were killed by an overdose of the anesthetic and the hearts were removed and weighed. The wet tissue to body weight ratios was calculated to assess the degree of congestion.

### Histopathological examination

The hearts were fixed in 10% buffered formalin. The tissues were embedded in paraffin, sectioned at 5 μm and stained with hematoxylin and eosin (H&E) for evaluation of histology, and Gomeri trichrome for distinguishing muscle and interstitial connective tissue. Myocardial fibrosis and necrosis was evaluated in each section of the heart tissue using a morphometric point-counting procedure [20]. Two persons graded the histopathological changes as 1, 2, 3, and 4 for low, moderate, high, and intensive pathological changes, respectively.

### Determination of lipid peroxidation in serum and myocardium

Malondialdehyde (MDA), a thiobarbiturate reactive substance, was measured as a marker for oxidative stress in serum and myocardial homogenates using a method prescribed by Satoh [21]. The lipid peroxide expressed as nanomole MDA production per gram heart tissue and nanomole per milliliter serum, were measured spectrophotometrically.

### Statistics

Data were presented as mean ± sem. One-way-ANOVA was used to make comparisons between the groups. If the ANOVA analysis indicated significant differences, a Student–Newman–Keuls post test was performed to compare the mean values between the treatment groups and the control. Any differences between groups were considered significant at P < 0.05.

## Results

### Phytochemical screening of O. basilicum extract

**Figure 1:**
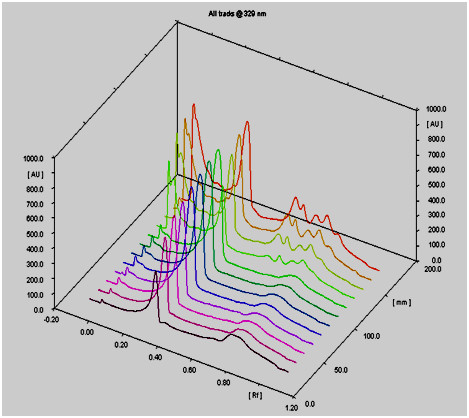
**Photodensitogram of the fluorescent HPTLC plate of rosmarinic acid (densitograms 1–8, from front to back, affording 0.5, 1, 1.5, 2, 2.5, 3, 3.5, and 4 μg rosmarinic acid) and *O. basilicum *ethanolic extract (densitograms from 9–14, affording 20, 40, 60, and 80 μg of the extract) depicted by TLC scanner 3(CAMAG) in which the X, Y and Z axis represents RF of each detected spot, height of the peaks (Spot’s density), and location on the plate, respectively.**

Preliminary phytochemical screening of hydroalcoholic extract of *O. basilicum* indicated the presence of flavonoids and phenolic compounds. The total amount of phenolic compounds determined as gallic acid was 53.66 mg/g extract (5.36%). The content of flavonoids in the extract was 18.65 mg quercetin equivalent/g of the extract (1.86%) by reference to a standard curve: y = 0.008x-0.068; r^2^ = 0.999. HPTLC analysis of the extract revealed the presence of rosmarinic acid with Rf value of 0.31 as the major compound. In order to quantitative determination of rosmarinic acid in the extract the fluorescent HPTLC plate was placed on TLC scanner and scanned under the UV 329 nm light. The resultant 3D graph was depicted on the basis of optical density (densitogram) (Figure 1). Further, the calibration curve was calculated automatically according to the densitogram data and rosmarinic acid in the hydroalcoholic extract of *O. basilicum* was calculated as 157.40 mg/g of *O. basilicum* extract by reference to a standard curve: y = 0.0092x-3.6997; r^2^ = 0.9947.

### *In vitro* antioxidant activity of O. basilicum extract

The ethanolic extract of *O. basilicum* was tested for its free radical scavenging effect on DPPH. DPPH was reduced concentration dependently by the extract. The free radical scavenging potency of the extract (IC50) was 22.24 μg/ml. This value was 9.7 μg/ml for rutin as a positive reference antioxidant.

### Effects of O. basilicum extract on electrocardiogram changes

**Figure 2:**
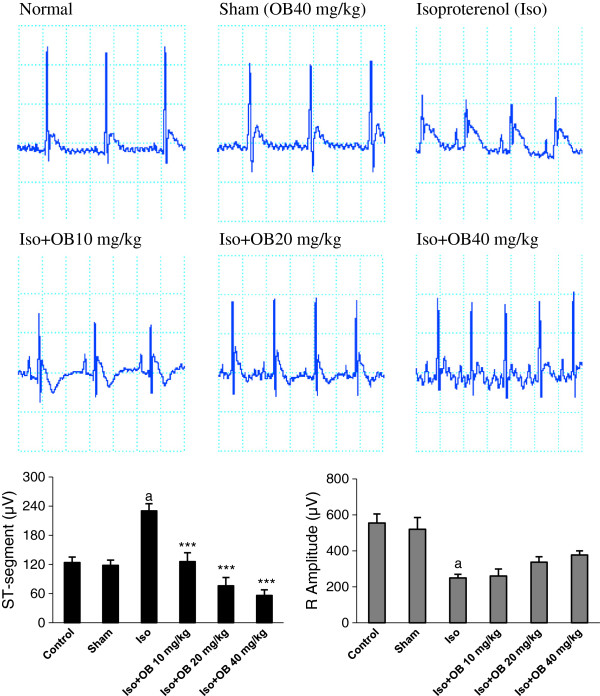
**Effect of the hydroalcoholic extract of *O. basilicum *(OB) on electrocardiographic patterns and changes (recorded from limb lead II) in normal control, isoproterenol alone injected (MI), and treated rats.** Values are mean ± sem (n = 6). ^**a**^P < 0.001 from respective control value; ^***^P < 0.001 as compared with isoproterenol treated group using one way ANOVA with Student-Newman-Keuls post hoc test.

The ECG from normal and treated rats has been shown in Figure 2. The normal control group and the rats that had received the extract alone (40 mg/kg; sham) showed normal patterns of ECG, whereas the rats treated with isoproterenol demonstrated a marked (p < 0.001) reduction in the R-amplitude along with a significant (p < 0.001) elevation of ST-segment, indicative of myocardial infarction (Figure 2). Treatment with all doses of the extract exhibited a marked and dose dependent inhibition of the elevation of ST-segment (p < 0.001). The extract also increased the R-amplitude dose dependently, but this increase did not attain a significant level (Figure 2).

### Effects of O. basilicum extract on hemodynamic responses

**Figure 3:**
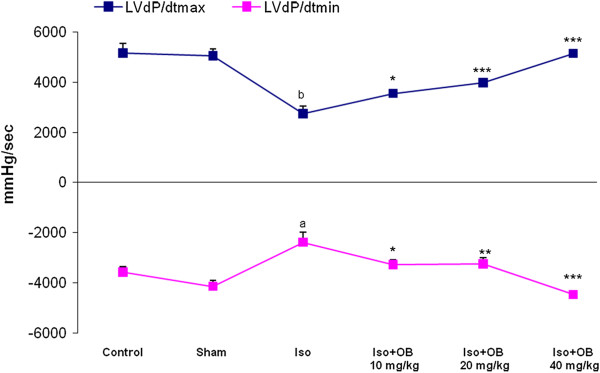
**Left ventricular maximal and minimal rates of pressure increase (LV *d*P/*d*t**_**max**_**; LV *d*P/*d*t**_**min**_**) in the control group and in the rats treated with isoproterenol alone (rats with myocardial infarction), *O. basilicum *extract alone (sham), and isoproterenol plus *O. basilicum *extract.** Iso: isoproterenol; OB: *O. basilicum* extract. Values are mean ± sem (n = 6). ^**a**^p < 0.05; ^**b**^p < 0.001 from respective control value; *p < 0.05, **p < 0.01, ***p < 0.001 as compared with isoproterenol treated group using one way ANOVA with Student-Newman-Keuls post hoc test.

Isoproterenol injection (animals with myocardial infarction) caused a significant decrease in the mean arterial blood pressure from 107 ± 6 mmHg in the normal control to 66 ± 6 mmHg (p < 0.01; Table 1). There was a considerable (p < 0.001) increase in the mean arterial blood pressure to 113±4 mmHg after treatment with 40 mg/kg of *O. basilicum* extract. The intraventricular pressure was measured to determine the degree of left ventricular responses to the isoproterenol injection. Isoproterenol significantly reduced the left ventricular systolic pressure (LVSP) from 125±7 mmHg in the normal control group to 72±9 mmHg (p < 0.001). All doses of the extract increased the LVSP and the increase with 20 and 40 mg/kg of the extract was prominent (p < 0.01; p < 0.001). There was almost a 3 fold elevation in the left ventricular end diastolic pressure (LVEDP) in the isoproterenol treated rats, thereby indicating left ventricular dysfunction. All three doses of the extract considerably (p < 0.001) improved the left ventricular function by lowering LVEDP from 21±1.8 mmHg in the rats with myocardial infarction to 5.8±1.1, 9.0±3.4, and 6.6±1.5 mmHg, respectively (Table 1). When compared with the normal control, the rats with left ventricular dysfunction (isoproterenol group) demonstrated a fall in the values of the left ventricular maximal and minimal rates of pressure (LV *d*P/*d*t_max_; LV *d*P/*d*t_min_, p < 0.001; p < 0.05; Figure 3) as well as a lower rate of pressure change at a fixed ventricular pressure (LV *d*P/*d*t/P, p < 0.01; Table 1). The same as the LVSP changes, the indices of myocardial contractility also showed marked and dose-dependent improvements (p < 0.05; p < 0.01; p < 0.001) by all doses of the extract.

**Table 1:** **Effects of the hydroalcoholic extract of *O. basilicum *on hemodynamic and left ventricular function in rats treated with isoproterenol**

**Groups**	**MAP (mmHg)**	**Herat rate (bpm)**	**LVSP (mmHg)**	**LVEDP (mmHg)**	**LV dP/dt/P (1/sec)**
**N = 6**					
**Control**	107±6	255±5	125±7	6.3±1.1	64±2.3
**Sham**	106±6	249±10	117±4	5.6±0.9	60±1.4
**Isoproterenol (Iso)**	66±6^**b**^	323±15^**a**^	72±9^**c**^	21±1.2^**c**^	38±9.1^**b**^
***O. basilicum *(10 mg/kg) + Iso**	78±6	285±19	95±4	5.8±1.1***	69±4.5**
***O. basilicum *(20 mg/kg) + Iso**	90±11	294±21	109±10**	9.0±3.4***	73±4.6***
***O. basilicum *(40 mg/kg) + Iso**	113±4***	312±16	127±3***	6.6±1.5***	95±2.8***

Data are expressed as mean ± sem. *MAP* Mean arterial pressure, *LVSP* left ventricular systolic pressure, *LVEDP* left ventricular end diastolic pressure. ^**a**^p < 0.05; ^**b**^p < 0.01; ^**c**^p < 0.001 from respective control value; *p < 0.05, **p < 0.01; ***p < 0.001 as compared with isoproterenol treated group using one way ANOVA with Student-Newman-Keuls post hoc test.

### Effects of O. basilicum extract on the heart weight to body weight ratio

In order to assess the extent of heart weight gain developed by the injection of isoproterenol, the heart weight to body weight ratio was determined (Table 2). The ratio was significantly higher in the isoproterenol treated rats (4.16 ± 0.05) compared with the normal control group (2.56 ± 0.02; p < 0.001). Oral treatment with 20 and 40 mg/kg of the *O. basilicum* extract exhibited a substantial reduction (p < 0.01 and p < 0.001, respectively) in the heart to body weight ratio (Table 2).

**Table 2:** **The effects of oral administration of hydroalcoholic extract of *O. basilicum *extracrt on Heart weight (HW) to body weight (BW) ratios, and on serum and myocardial malondialdehyde (MDA) levels**

**Groups (n = 5-6)**	**HW/BW ratio (g/kg)**	**Serum MDA (nmol/ml)**	**Myocardium MDA (nmol/mg)**
**Control**	2.56 ± 0.02	5.4 ± 0.48	1.6 ± 0.13
**Isoproterenol**	4.14 ± 0.05^a^	13.7 ± 0.2^**a**^	3.9 ± 0.36^a^
***O. basilicum (*10 mg/kg) + Iso**	4.06 ± 0.05	7.9 ± 1.7^***^	2.3 ± 0.19^***^
***O. basilicum *(20 mg/kg) + Iso**	3.70 ± 0.11**	7.1 ± 0.4^***^	1.7 ± 0.03^***^
***O. basilicum *(40 mg/kg) + Iso**	3.43 ± 0.025***	4.4 ± 0.4^***^	1.4 ± 0.01^***^

Data are expressed as mean ± sem (n = 5-6). ^a^p < 0.001 from respective control value; *p < 0.05; **p < 0.01; ***p < 0.001 as compared with isoproterenol treated group using one way ANOVA with Student-Newman-Keuls post hoc test.

### Histopathological examination of the cardiac tissues

**Figure 4:**
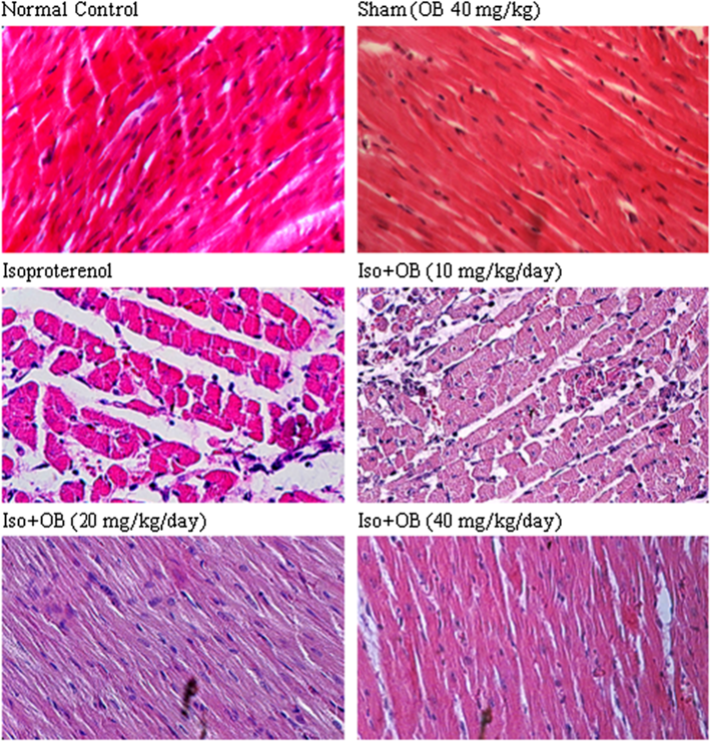
**Photomicrographs of sections of rat cardiac apexes.** Heart tissue of a rat subcutaneously injected with isoproterenol alone shows intensive cardiomyocyte necrosis and increased edematous intramuscular space. Treatment with *O. basilicum* extract demonstrates a marked improvement. Iso: isoproterenol; OB: *O. basilicum* extract. H&E (40 M).

**Figure 5:**
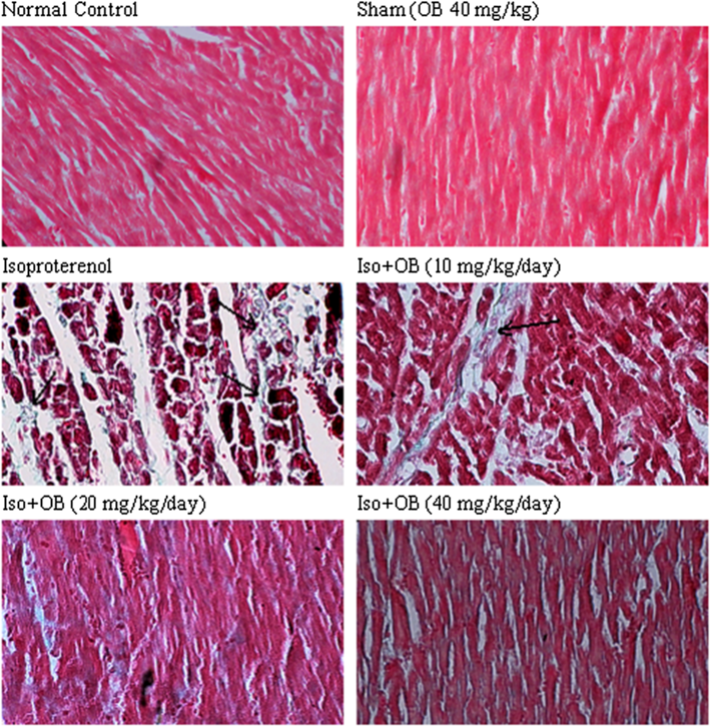
**Heart tissue of a rat treated with isoproterenol shows an intensive fibrosis (blue spots are shown by arrows) and angiogenesis.** Pretreatment with *O. basilicum* extract significantly prevented the fibrosis. Iso: isoproterenol; OB: *O. basilicum* extract. Gomeri's one-step Trichrom method (40 M).

**Figure 6:**
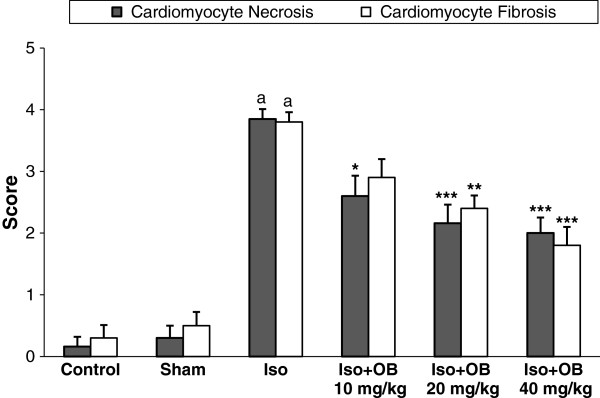
**The effects of oral administration of hydroalcoholic extract of *O. basilicum *extracrt on grading of histopathological changes in rats’s cardiac apex tissues (scors 1, 2, 3, and 4 show low, moderate, high and intensive pathological changes, respectively).** Data are expressed as mean ± sem (n = 5-6). ^a^p < 0.001 from respective control value; *p < 0.05; **p < 0.01; ***p < 0.001 as compared with isoproterenol treated group using one way ANOVA with Student-Newman-Keuls post hoc test.

In the normal control group, myocardial fibers were arranged regularly with clear striations. No apparent degeneration or necrosis was observed (Figure 4). Histological sections of the isoproterenol treated hearts showed widespread subendocardial necrosis, hypertrophia, and abundant fibroblastic hyperplasia along with increased edematous intramuscular space (Figure 4 and Figure 5). All the three doses of *O. basilicum* extract significantly prevented myocardial necrosis and fibrosis. The *O. basilicum* extract with doses of 10, 20, and 40 mg/kg reduced the isopreterenol-induced necrosis and edematous dose dependently by 33% (p < 0.05), 44% (p < 0.001), and 48% (p < 0.001), respectively as shown in Figure 6. The cardiomyocyte fibrosis was also reduced by 24%, 37% (p < 0.01), and 53% (p < 0.001; Figure 6).

### Effects of O. basilicum extract on lipid peroxidation

To determine the lipid peroxidation, MDA levels were measured in serum and myocardial homogenates. Both serum and heart MDA levels were considerably increased (p < 0.001) in isoproterenol injected rats (MI group) in comparison with normal control (Table 2). Treatment with *O. basilicum* extract markedly diminished the serum MDA levels by 43-68% and myocardial MDA levels by 41-64% (p < 0.001; Table 2).

## Disscussion

*Ocimum basilicum L.*, known as basil, is a culinary herb that is used in Asian and Italian cuisine. *O. basilicum* are being used traditionally for the treatment of gastrointestinal disorders [2,3], diabetes [22], headaches, cardiovascular and neurodegenerative disorders [23], and as anti-inflammatory [24]. An endothelium-dependant vasorelaxant and anti-platelet aggregation activities of an aqueous extract from *O. basilicum* have also been reported [25]. In the present study, we investigated the therapeutic efficacy of the hydroalcoholic extract of leaves of the plant in rats with myocardial infarction. A subcutaneous injection of a high dose of isoproterenol resulted in electrocardiographic, hemodynamic, and structural changes in the heart very similar to that occurs in patients with myocardial infarction. Isoproterenol elevated the ST-segment in the ECG (diagnostic of myocardial infarction) and treatment with the hydroalcoholic extract of *O. basilicum* considerably amended the ECG pattern by suppressing the ST-segment elevation. Isoproterenol injection significantly decreased the arterial pressure indices, contractility (LVdP/dt_max_) and relaxation (LVdP/dt_min_), and increased the left ventricular end-diastolic pressure (LVEDP). *O. basilicum* substantially recovered the arterial pressure and improved the left ventricular performance along with a simultaneous reduction in the LVEDP.

Myocardial infarction is a leading cause of mortality and morbidity worldwide. Left ventricular dysfunction and heart failure are common following a myocardial infarction and various treatments are given to prevent the complications. Today, herbal medicine as an adjunctive therapy has been considered increasingly in the treatment of myocardial infarction. There are considerable epidemiological evidences suggesting that consumption of fruits and vegetables, particularly green leafy vegetables is associated with a lower risk for coronary heart diseases [26,27]. The beneficial effects of vegetables and fruits could be due to the presence of compounds with antioxidant properties. The formation of reactive oxygen species plays a key role in cardiac pathophysiology. Therefore, by targeting oxidative stress the treatment of myocardial infarction can be substantially improved. In fact, the acute phase of myocardial necrosis induced by isoproterenol has been linked to generation of highly cytotoxic reactive oxygen [12]. Several studies have reported that *O. basilicum* has strong antioxidant properties and individual phenolic acid composition strongly influences the antioxidant capacity [28,29].

Our preliminary phytochemical finding in this study revealed the presence of phenolic compounds and flavonoids in the hydroalcoholic extract obtained from *O. basilicum* leaves. Ever since oxidative stress related conditions like isoproterenol induced damages are generated via an imbalance between the production of oxidants and occurrence of antioxidant defenses, searching for flavonoids and other phenolic compounds in *O. basilicum* extract is of value for quantifying the putative role of them as antioxidant agents, for confronting this oxidative stress. Considering the findings of the study, radical scavenging activity of the extract was in line with the high phenolic and flavonoid contents of *O. basilicum* extract. Moreover, rosmarinic acid with 15.74% presence was identified as a major phenolic derivative of the *O. basilicum* extract in the present study. Rosmarinic acid which is commonly found in species of the lamiaceae such as culinary herbs has a number of interesting biological activities, e.g. antiviral, antibacterial, antiinflammatory and antioxidant [30]. The results of the present study show a considerable *in vitro* (IC_50_ = 22.24 μg/ml compared with rutin with IC_50_ = 9.7 μg/ml) and in vivo antioxidant activity of *O. basilicum* extract, so that the extract diminished the myocardial and serum lipid peroxidation by 41-68%. Besides, it has been suggested that the observed scavenging activity of the extract could be assigned to the hydrogen-donating capacity of the phenolic and flavonoid components, present in sizeable amounts of *O. basilicum* extract, signifying the potential role of them in preventing the isoproterenol induced damages in this study. Accordingly, developing an efficient herbal remedy is reliant to a superior notion of the relation between chemical constituents and biological properties of the plant material.

Eventually, we conclude that short term administration of hydroalcoholic extract of *O. basilicum L.* (basil) leaves potently protects the myocardium against isoproterenol induced infarction and suggest that the cardioprotective effect of the extract could be related to its antioxidant activities.

## Competing interests

The authors’ declare that they have no competing interests.

## Authors' contribution

FF: Introduced the plant and all phytochemial analysis have been done under her supervision. AM: Prepared the plant material and the extract and carried out animals grouping and handling. AK: Carried out the hemodynamic experiments. SH: Carried out the phytochemical experiments. HS: Carried out the biochemical experiments. MH: Accompanied Amin Matlobi in animal handling and MI induction. NM-D: Histopathological works and interpretations. AG: Supervising and directing the project, carried out the data analysis and interpretations and prepared the manuscript. All authors read and approved the final manuscript.
